# IDH1 Mutation Impacts DNA Repair Through ALKBH2 Rendering Glioblastoma Cells Sensitive to Artesunate

**DOI:** 10.3390/biomedicines13061479

**Published:** 2025-06-16

**Authors:** Olivier Switzeny, Stefan Pusch, Markus Christmann, Bernd Kaina

**Affiliations:** 1 Institute of Toxicology, University Medical Center, 55131 Mainz, Germany; olivier.switzeny@outlook.com (O.S.); mchristm@uni-mainz.de (M.C.); 2 Department of Neuropathology, Institute of Pathology, University Hospital Heidelberg, 69120 Heidelberg, Germany; s.pusch@dkfz-heidelberg.de; 3 Clinical Cooperation Unit Neuropathology (B300), German Consortium for Translational Cancer Research (DKTK), German Cancer Research Center (DKFZ), 69120 Heidelberg, Germany

**Keywords:** artesunate, IDH1, glioblastoma, temozolomide, ALKBH2, DNA repair

## Abstract

**Background**: Isocitrate dehydrogenase 1 and 2 (IDH1 and IDH2) are enzymes that catalyze the oxidative decarboxylation of isocitrate to alpha-ketoglutarate (α-KG), which is essential for many metabolic processes, including some steps in DNA repair. In tumors, notably in gliomas, *IDH1* and *IDH2* are frequently mutated. The mutation found in different cancers is functionally active, causing, instead of α-KG, the formation of 2-hydroxyglutarate (2-HG), which inhibits α-KG-dependent enzymes. Gliomas harboring mutated *IDH1/2* show a better prognosis than *IDH1* wild-type (wt) tumors of the same grade, which might result from the inhibition of DNA repair functions. A DNA repair enzyme dependent on α-KG is alkB homolog 2 (ALKBH2), which removes several lesions from DNA. These findings prompted us to investigate the response of glioma cells to artesunate (ART), a plant ingredient with genotoxic and anticancer activity currently used in several trials. **Materials and Methods**: We used isogenic glioblastoma cell lines that express IDH1 wild-type or, based on a TET-inducible system, the IDH1 mutant (mt) protein, and treated them with increasing doses of artesunate. We also treated glioblastoma cells with 2-HG, generated ALKBH2 knockout cells, and checked their sensitivity to the cytotoxic effects of artesunate. **Results**: We show that the cell-killing effect of ART is enhanced if the IDH1 mutant (R132H) is expressed in glioblastoma cells. Further, we show that 2-HG imitates the effect of IDH1mt as 2-HG ameliorates the cytotoxicity of ART. Finally, we demonstrate that the knockout of ALKBH2 causes the sensitization of glioblastoma cells to ART. **Conclusions**: The data indicate that ALKBH2 protects against the anticancer effect of ART, and the mutation of IDH1/2 commonly occurring in low-grade gliomas sensitizes to ART via an ALKBH2-dependent mechanism. The data support the use of ART in the therapy of *IDH1/2*-mutated cancers both in combination with chemotherapy and adjuvant treatment.

## 1. Introduction

Diffuse gliomas are the most common subtype of primary brain tumors, which are aggressively growing, highly invasive, and neurologically destructive with a dismal prognosis despite standard therapies [1]. They are classified into low- and high-grade (WHO grade 4) tumors. An important molecular diagnosis marker is the genetic mutation in either isocitrate dehydrogenase 1 (*IDH1*) or isocitrate dehydrogenase 2 (*IDH2*), which is common in grade 2 and 3 gliomas and defining astrocytoma and oligodendroglioma [1,2]. *IDH*-mutated gliomas are characterized by slower growth and a significantly better prognosis than *IDH* non-mutated tumors, even if tumors from the same grade were compared [3]. The alterations in *IDH1* or *IDH2* are dominant gain-of-function mutations, which are considered to be a prognostic marker for gliomas [4].

*IDH1/2* mutations were not only found in gliomas but also in other tumors such as acute myeloid leukemia (AML), myelodysplastic syndrome, myeloproliferative neoplasm, cholangiosarcoma, enchondroma, chondrosarcoma, and other solid cancers [5,6]. Wild-type IDH1 and 2 catalyze the oxidative decarboxylation of isocitrate to alpha-ketroglutarate (α-KG), also known as 2-oxoglutarate. Mutations in either *IDH1* or *IDH2* are heterozygous. Both IDH1 and IDH2 form a homodimer to exert their catalytic function. Therefore, in a tumor cell carrying a single *IDH* mutation, the majority of dimers harbor the mutant protein performing the altered function [7]. Nearly all identified mutations represent a single amino acid missense mutation in IDH1 at arginine 132 (R132) or the analogous residue (R172) in IDH2 [8]. Both *IDH1* R132H and R132C mutations involve a conversion of a CpG dinucleotide to TpG on opposite strands of the *IDH* R132 codon, which likely results from a spontaneous deamination event [9].

*IDH1/2* mutations result in the abnormal production of 2-hydroxyglutarate (2-HG) instead of α-KG. Notably, 2-HG was found to inhibit the enzymatic function of many α-KG-dependent dioxygenases, including histone (Jumonji domain-containing histone-lysine demethylases) and DNA demethylases (Ten-eleven translocation family), causing widespread changes in histone and DNA methylation and potentially promoting tumorigenesis [10,11]. The 2-HG concentration in patient-derived IDH1-mutated tumor cells ranged from 5 to 35 mM [12,13]. It is reasonable to suppose that the oncometabolite 2-HG contributes to malignant transformation and the therapeutic response of *IDH*-mutated cancer cells. As outlined above, glioma patients carrying an *IDH* mutation have a significantly prolonged progression-free survival and overall survival compared to *IDH* wild-type glioma patients [8,14,15].

Since the discovery of *IDH* mutations, great efforts have been made to exploit them for an improved treatment. Thus, *IDH*-mutated tumor cells in vitro and low-grade gliomas were shown to be more sensitive to chemotherapy by temozolomide [16,17] and radiation therapy [18,19]. However, data reported in preclinical studies are conflicting. Thus, it was shown that mutant IDH1 leads to TMZ resistance by upregulating homologous recombination [20], while others found that IDH mutations suppress homologous recombination [21]. Again, other authors could not find any differences between the IDH mutant and the corresponding wild-type as to temozolomide sensitivity [19,22]. Radiation decreased the viability of IDH-mutated cells more than wild-type counterparts, and increased ROS levels were found in the mutated cells [19]. ROS levels were always higher in IDH-mutated cells after chemo and/or radiation treatment, even in untreated cells [19,23,24,25,26]. Increased ROS levels are believed to contribute to the observed phenotypes. The product of the IDH wild-type protein, α-KG, is an essential cofactor of the DNA repair enzyme ALKBH2, which removes several damaged bases from DNA via oxidative dealkylation [27]. Based on this, we hypothesized that IDH mutant cells defective in this repair pathway respond accordingly to genotoxic stress.

A natural compound inducing oxidative stress is artesunate (ART). This is a semi-synthetic derivative of the herbal *Artemisia annua* ingredient artemisinin, which has been used for centuries in traditional Chinese medicine (TCM). Due to the very low water solubility of the natural compound, a number of derivatives have been synthesized, including ART, which is currently used as an antimalaria drug because of its potent activity against the chloroquine-resistant pathogen *Plasmodium falciparum* [28]. ART also has anticancer activity, which was extensively studied in different experimental systems, including gliomas [29]. Several trials have been conducted to demonstrate safety and anticancer activity in patients [30]. This makes it a reasonable candidate for a cancer chemotherapeutic agent inhibiting metastasis [31], cancer-related signaling pathways [32,33,34], and angiogenesis [35,36]. ART is an endoperoxide that generates intracellular ROS, leading to 8-oxoG and other oxidative DNA damages [37]. Furthermore, ART causes lipid peroxidation, which gives rise to the formation of 1,N^6^-ethenoadenine and other products that are substrates for ALKBH2 [37,38,39]. ART has also been shown to induce DNA double-strand breaks, activate the ATM/ATR-dependent damage response [37], and inhibit homologous recombination, thus enhancing the response of glioma cells to temozolomide [40].

Here, we report on the impact of IDH1mt on ART-induced cytotoxicity in glioma cells. To exclude variable cellular background and long-term secondary effects resulting from 2-HG, we used an isogenic cell system based on an inducible vector encoding the mutant protein. We show that IDH1mt (R132H) cells are more sensitive to ART than the wild-type. Sensitization to ART was also provoked by 2-HG produced in IDH1mt cells. Furthermore, we show that ALKBH2 deficiency (knockout) sensitized cells to ART. The data strongly indicate that ALKBH2-mediated DNA repair is involved in the defense against ART-induced DNA damage and propose ART as a therapeutic supplemental agent notably for IDH1/2-mutated gliomas and presumably also other cancers displaying this mutation.

## 2. Materials and Methods

### 2.1. Cell Culture and Drug Treatment

The cell lines used were described previously [41]. The TET-inducible LN319 glioblastoma cell lines expressing either wt IDH, R132H mt IDH1, or the vector control were characterized and described previously [42]. LN319 cells were maintained in DMEM (Gibco, Thermo Fisher Sci. Inc., Waltham, MA, USA) supplemented with 5% TET-system-approved fetal bovine serum (Takara Bio, London, UK) in the presence of geneticin (4 mg/mL) and blasticidin (80 µg/mL). LN18, T98G, and the corresponding ALKBH2 knockout clonal cell lines (T98G D7 and LN18 G5) were maintained in DMEM supplemented with 5% fetal bovine serum (Gibco).

### 2.2. Generation of Knockout and Western Blots

ALKBH2 knockout was performed using the ALKBH2 CRISPR/Cas9 KO Plasmid (#sc-407363, Santa Cruz Biotechnology), which consists of three plasmids, each harboring a different gRNA that target the second or third exon of the *ALKBH2* gene and a GFP marker on the plasmids. LN18 and T98G cells were transfected using Effectene (Qiagen, Hilden, Germany) and collected after 48 h. Cells designated “mock” were transfected with the vector only. GFP-positive cells were single cells sorted on a FACS Aria III SORP cell sorter (BD Biosciences, Heidelberg, Germany) and seeded into 96-well plates. Cell clones were expanded, and successful ALKBH2 knockout was assayed by determining the level of ALKBH2 protein using the ALKBH2 rabbit antibody from SAB Biotech (Greenbelt, Maryland, USA). The mouse antibody against IDH1 R132H detecting specifically the mutant protein was from Dianova (Hamburg, Germany) and the mouse antibodies detecting ß-actin and HSP90 were from Santa Cruz Biotechnology (Dallas, TX, USA). ß-actin and HSP90 served as loading control. Western blots were performed with extracts of exponentially growing cells essentially as previously described [43].

### 2.3. Cytotoxicity Assays

For cell viability tests, cells were seeded into 96-well plates, allowed to attach for at least 24 h, and exposed to ART for up to 120 h. The medium was not replaced. Viability was measured using the metabolic MTT assay as previously described [43]. Absorbance was measured at 570 nm on a Tristar LB942 reader (Berthold Tech., Bad Wildbad, Germany). Untreated controls were set to 1. Apoptosis and necrosis were measured by flow cytometry of annexin V and propidium iodide (AV/PI) stained cells essentially as previously described [43].

### 2.4. Statistics

Data are the mean of at least three independent experiments ± S.D. Data were pooled and analyzed by the two-way ANOVA with the Tukey post hoc test.

## 3. Results

### 3.1. IDH1 Mutation Renders Cells More Sensitive to Artesunate

**Figure 1:**
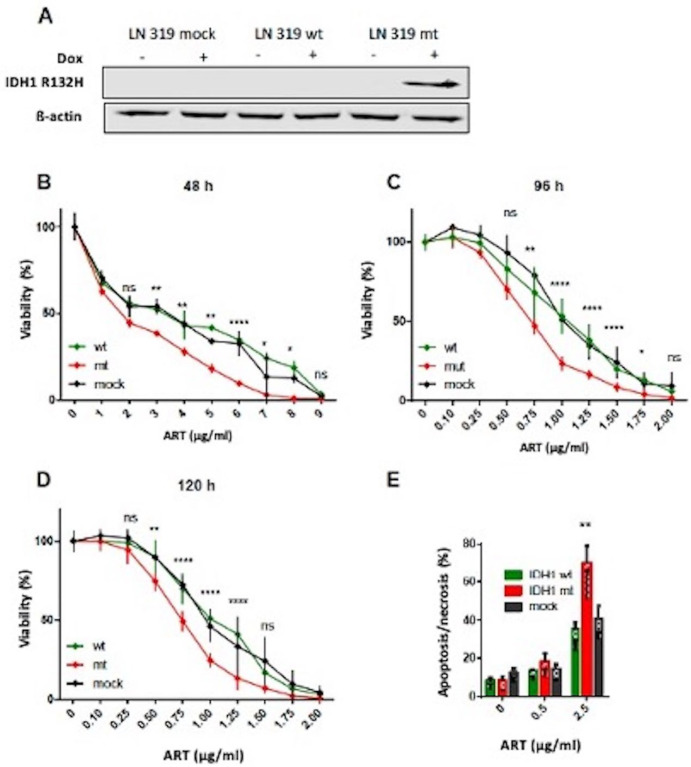
Responses of LN319 (wt) cells and cells expressing IDH1 wild-type (mock) and IDH1 mutant protein. (**A**) Western blot. Dox, doxycycline treatment. (**B**–**D**) Cells were exposed to increasing doses of ART and cell viability was measured by MTT assay at various time points after exposure. (**E**) Cell death by apoptosis and necrosis were determined by AV/PI flow cytometry. The yield of necrosis (marked with hatching) was <30%. Data are the mean of at least three independent experiments ± S.D. Significance levels compared to control: ns, not significant; * *p* < 0.05, ** 0.1, *** 0.01, **** 0.001. It should be noted that viability and cell death through apoptosis/necrosis cannot be directly compared quantitatively, as viability is a metabolic assay affected by other parameters (including proliferation inhibition) than annexin/PI staining.

*IDH1* R132H mutation led to epigenetic reprogramming of the genome, which resulted in the glioma CpG island methylator phenotype (G-CIMP). Immortal astrocytes expressing IDH1 R132H showed a higher DNA methylation pattern from passage 15 onwards compared to the parental cell line [44]. To avoid this long-term effect, we expressed IDH1wt, mutant IDH1 R132H (IDH1mt), and vector control in LN319 glioblastoma cells in a TET-inducible system. The induced expression of IDH1mt following doxycycline treatment for 24 h was confirmed by Western blot analysis (Figure 1A). The expression of mutated IDH1 did not influence the cell’s proliferation rate. Using the MTT assay, we found that cells expressing the IDH1mt protein were more sensitive to ART than the IDH1wt and vector control (Figure 1B,C,D for 48, 96, and 120 h exposure, respectively). We should note that both proliferation inhibition and cell death were variables impacting the metabolic assay.

To further substantiate the findings, we measured the yield of cell death (apoptosis and necrosis) by annexin V/PI flow cytometry following ART treatment of IDH1mt- and IDH1wt-expressing cells and the corresponding vector control. After 120 h of treatment, IDH1mt displayed significantly higher levels of apoptosis and necrosis (Figure 1E) compared to IDH1wt and vector control. The data led us to conclude that the expression of IDH1 R132H sensitizes glioblastoma cells to ART-induced cytotoxicity.

### 3.2. 2-Hydroxyglutarate Increases the Toxicity of Artesunate

**Figure 2:**
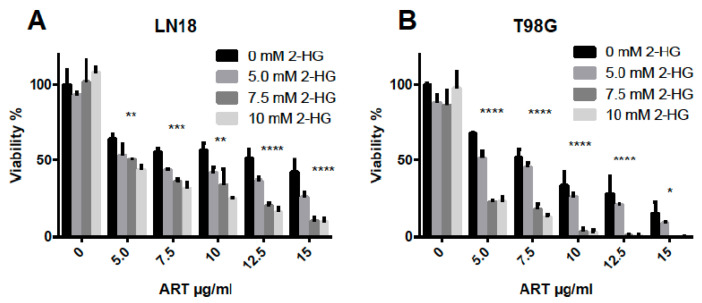
Effect of artesunate on the viability of LN18 (**A**) and T98G (**B**) glioblastoma cells. Viability was measured by the MTT assay 96 h after the onset of treatment. Data are the mean of at least three independent experiments ± S.D. Significance levels compared to control: * *p* < 0.05, ** 0.1, *** 0.01, **** 0.001.

IDH1 mutant cells and the corresponding tumors are characterized by a high level of 2-HG, which is produced by the mutant enzyme instead of α-KG. Notably, 2-HG competitively inhibits several α-KG-dependent enzymes, including the DNA repair enzyme ALKBH2 [45]. Here, we hypothesized that α-KG-dependent repair functions such as ALKBH2 could be involved in mediating ART resistance. If true, we anticipated that 2-HG would be able to imitate the effect of IDH1mt and analyzed whether IDH1wt cells display increased ART-induced cytotoxicity following 2-HG treatment. The physiological 2-HG concentrations in human *IDH1*mt gliomas have been quantified, ranging between 5 and 35 µmol per gram of tumor, corresponding to 5–35 mM assuming a tissue density of 1.05 g/mL [12,46]. We therefore used 2-HG concentrations within this range for these experiments. Notably, 2-HG did not affect cell growth and viability up to a concentration of 10 mM (Figure 2). We exposed LN18 and T98G cells to increasing doses of 2-HG 1 h before ART treatment and measured the cell viability. In both cell lines, 2-HG significantly augmented the ART-induced cytotoxicity (Figure 2A,B). The data demonstrate that 2-HG enhances the sensitivity of cells to ART, supporting the role of ALKBH2 in defense against ART-induced cell-killing effects.

### 3.3. ALKBH2 Knockout Causes an Increase in Artesunate Toxicity

**Figure 3:**
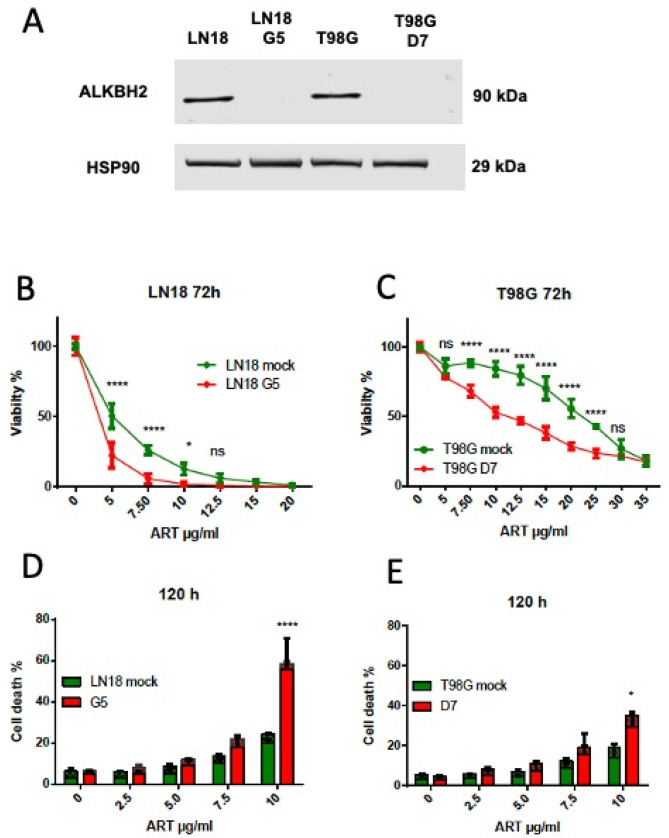
Response of ALKBH2 ko cells to artesunate: (**A**) Expression of ALKBH2 protein in wild-type (mock) and ko cells (the lines G5 and D7). (**B**,**C**) Cytotoxicity of artesunate in LN18 and T98G wild-type and ko cells treated with artesunate. (**D**,**E**) Cell death (apoptosis and necrosis) as measured by A/PI of LN18 and T98G cells following treatment with ART. Data from at least three independent experiments are pooled. Significance levels: ns, not significant; * *p* < 0.05, **** 0.001.

To further demonstrate the functional role of ALKBH2 in repairing ART-induced DNA damage, we generated ALKBH2 knockout cells in the human GBM cell lines LN18 and T98G, using a CRISPR/CAS approach. ALKBH2 knockout in the cell clones was verified in Western blot experiments. For each cell line, an ALKBH2 knockout clone was expanded (designated as LN18-G5, T98G-D7; for protein expression, see Figure 3A) and used for further experiments.

As shown in Figure 3B,C, the viability of ART-treated ALKBH2 ko cells was significantly reduced compared to the control expressing ALKBH2. Furthermore, the yield of apoptosis/necrosis (total cell death) was significantly enhanced in the knockout (Figure 3D,E). Overall, the data strongly support the notion that ALKBH2 is involved in repairing cytotoxic DNA lesions induced by ART, and mutant IDH1 mediates the hypersensitization of cells to the cytotoxic effect of ART via 2-HG.

## 4. Discussion

Standard treatment for grade 3 and 4 gliomas typically includes radiotherapy and genotoxic chemotherapy [47,48], and mutant IDH status is a significant prognostic marker [8,14,15]. The better survival and the improved responsiveness to alkylating chemotherapy of patients with mutant IDH are thought to be, at least in part, related to the metabolite formed by the mutated enzyme, 2-HG, which competes with α-KG and thus inhibits enzymes that are functionally dependent on this metabolite. One of these is the ALKBH2 repair protein, which is a DNA repair dioxygenase that removes DNA alkylation lesions (1-methyladenine, 3-methylcytosine, and others) [27]. The enzyme uses an oxidative demethylation mechanism, requiring α-ketoglutarate. It converts the methyl group attached to DNA bases into formaldehyde, thus reversing the damage [49,50]. The contribution of the different DNA methylation adducts that are subject to repair by ALKBH2 to the cytotoxic effect of methylating drugs such as temozolomide and procarbazine, inducing the main killing lesion is O^6^-methylguanine (for review, see [51]), remains to be established. It was shown, however, that ALKBH2 knockdown [52] or ALKBH2 inhibition by 2-HG in IDH mutant cells [27] increases the cell’s sensitivity to temozolomide. This indicates that one or several of the DNA methylation lesions repaired by ALKBH2 are cytotoxic. It is of interest that ALKBH2 is frequently upregulated in glioblastomas, and patients with high expression levels have a reduced overall survival [45], supporting this view and suggesting ALKBH2 as a potential prognostic biomarker in gliomas. Overall, the data strongly suggest that the anticancer effect of temozolomide (and other methylating drugs) is determined by both MGMT and ALKBH2 [51].

Other adducts produced by ROS generators and repaired by ALKBH2 are etheno-adducts, among them 1,N^6^-ethenoadenine and 3,N^4^-ethenocytosine [53]. The exact pathway of intracellular etheno-adduct formation is unclear, but it is accepted that the lesions can be generated by oxidative stress causing lipid peroxidation and the formation of trans-4-hydroxy-2-nonenal, malonaldehyde and crotonaldehyde, which are highly DNA-reactive species [54,55,56].

The phytochemical artemisinin and its semisynthetic derivative ART are, due to a cleavable peroxide bridge, primarily ROS generators. ART is clearly cytotoxic and genotoxic, inducing DNA damage, which is repaired via different pathways such as base excision repair, homologous recombination, and non-homologous end-joining [57]. In glioblastoma cells, ART was shown to induce oxidative DNA damage and DNA double-strand breaks that accumulate during the treatment period and finally activate the ATM/ATR axis and DNA damage-dependent pathways causing cell cycle inhibition and apoptotic cell death [37]. ART is also an inducer of ferroptosis [58] and autophagy [59] and exerts additive and synergistic action with different anticancer drugs, including TMZ [40,60]. Further, it was shown to downregulate RAD51 expression, resulting in the inhibition of DNA repair through homologous recombination and drug-induced cellular senescence [40]. Recently, we have shown that the phytochemical also exerts senolytic activity on glioblastoma cells [61], which is important as senescence is triggered by radiation and anticancer drugs, including temozolomide [43]. Since senescent cells drive tumorigenesis through the senescence-associated secretory phenotype (SASP) [62], which appears to play a critical role in glioblastoma [63], their eradication appears to be important. ART also provokes antiproliferative effects by activating the ROS-triggered AMPK-mTOR axis [64]. Interestingly, combined with a low dose of metformin, it reduced the viability, migration, and invasion capacity of glioblastoma cells [65]. Taken together, these data indicate that ART can be considered a mildly cytotoxic anticancer agent and, at the same time, an enhancer of the anticancer activity of temozolomide and other genotoxic drugs.

With this background information at hand, we addressed the question of whether the mutation of IDH1, via the inhibition of the α-KG-dependent dioxygenase ALKBH2, has an impact on the cell-killing effect of ART. We found that the expression of IDH1mt (using a TET-inducible system with an identical cellular background) renders the cells sensitive to ART, and pretreatment with 2-HG has the same effect. We further revealed that ALKBH2 knockout glioblastoma cells are more sensitive to ART than cells expressing ALKBH2. Overall, the data strongly support the notion that critical lesions induced by ART are repaired by ALKBH2, which is inactive in IDH1mt cancer cells due to 2-HG formation.

It should be mentioned that intracellular ROS produced by ART affects not only DNA, but also RNA, proteins, organelles, and membrane components. However, the main target responsible for cytotoxicity appears to be the DNA, as revealed by the previously reported hypersensitivity of DNA repair-defective mutants [57]; the activation of the ATM, ATR, CHK1 and CHK2 damage response [37]; and the sensitivity of ALKBH2-defective cells to ART reported here. ALKBH2 does not repair 8-oxo-guanine, however, it targets several ethenoadducts, which are formed via ROS-induced lipid peroxidation. Therefore, we hypothesize that non-repaired ART-induced ethenoadducts such as 1,N^6^-ethenoadenine blocking replication trigger the IDH1mt-specific response. Actually, ART provokes an S-phase and G2 block, which is more prominent when ALKBH2 is not expressed. Overall, the data reveal that cells harboring mutant IDH1 are hypersensitive to ART, which makes the inclusion of the phytochemical in therapeutic regimens an attractive strategy.

It should be noted that not only gliomas but also other cancers have been reported to involve an IDH mutant status [6], thus producing 2-HG that might make them vulnerable to ART. The phytochemical has been investigated in a panel of clinical phase 1 and 2 trials in various tumor types other than glioblastoma during the past years, e.g., metastasized breast cancer, cervical carcinoma, and non-small cell lung cancer (recently reviewed in [30]). In all cases, ART was administered together with other chemotherapeutics, which makes it difficult to assess the benefit. However, a series of case reports on different tumor entities, stating that artemisinin treatment improved the patient’s quality of life, can be taken into account to underline the clinical benefit of artemisinin and its derivatives. Importantly, ART has been shown to be able to cross the blood–brain barrier [66] and therefore is highly effective also in the treatment of cerebral malaria [67].

What are the therapeutic implications for gliomas? Until now, very limited clinical trials have been conducted. Thus, in a case series including low- and high-grade gliomas, patients were treated with ART (100 mg twice daily) alongside temozolomide and/or CCNU-based chemotherapy. The study reported that artesunate is a well-tolerated supportive agent, both in combination with chemotherapy and in long-term maintenance treatment [30]. Because of the small number of patients, conclusions cannot be drawn as to the benefits for IDHmt compared to IDHwt gliomas. Nevertheless, the causal link between IDHmt, high intracellular level of the oncometabolite 2-HG, and ALKBH2 inactivation favor the use of ART especially in the therapy of IDHmt tumors, both in combination with conventional treatment protocols and as a post-treatment supplement. Since radiation and temozolomide are potent inducers of cellular senescence in glioma cells [43,68], and ART bears senolytic activity [61], the compound also appears to be useful as a senotherapeutic for clearing therapy-induced senescent cells in both IDHmt and wt tumors, including glioblastomas. It should be noted that ART has been included in the CUSP9v3 protocol, a combination of nine repurposed drugs aimed at treating recurrent glioblastoma [69]. A phase Ib/IIa clinical trial [NCT02770378] reported that 30% of participants remained alive and disease-free after over four years, indicating potential efficacy. However, the small sample size and the mixture of drugs limit conclusions as to the contribution of ART. While the findings are promising, it is clear that comprehensive clinical trials are required to establish the safety and efficacy of ART in low- and high-grade glioma treatment. Finally, it should be noted that IDH is mutated in some other solid cancers [6] associated with high 2-HG levels [12], raising the question of whether ART is also effective in these tumor entities.

## 5. Conclusions

The inclusion of ART in the therapy of malignant glioma appears to be feasible and well tolerated by patients. The new preclinical findings reported here favor the use of ART, especially in the therapy of IDH-mutated tumors, which might be combined with conventional treatment protocols such as PCV, radiotherapy, and temozolomide. As ART bears senolytic activity, is well tolerated, and is already widely used in malaria therapy, it can be considered a repurposed drug useful not only in combination with temozolomide but also after a genotoxic therapy interval for clearing therapy-induced senescent cells in IDH1-mutated and non-mutated gliomas.

## Data Availability

Data can be obtained by request.
